# Evaluating Increment and Decrement Stimuli Responses in Patients with Glaucoma Using Virtual Reality–Based Perimetry

**DOI:** 10.1016/j.xops.2025.100929

**Published:** 2025-08-28

**Authors:** Albert Xu, Allen Khudaverdyan, Corinne Shiu, Michael Deiner, Murtaza Saifee, James J. Blaha, Benjamin T. Backus, Yvonne Ou

**Affiliations:** 1 Department of Ophthalmology, University of California, San Francisco, California; 2 Davidorf Eye Group, West Hills, California; 3 Vivid Vision Inc, San Francisco, California; 4 College of Optometry, State University of New York, New York

**Keywords:** Glaucoma, ON/OFF pathway, Perimetry, Virtual reality, Increment/decrement stimuli

## Abstract

**Objective:**

To evaluate increment and decrement stimuli responses in glaucoma using a virtual reality–based perimetric system, Vivid Vision Perimetry (VVP) and to compare these findings with conventional perimetry.

**Design:**

This is a cross-sectional study involving participants with diagnosed perimetric glaucoma, preperimetric glaucoma, and glaucoma suspect or ocular hypertension.

**Participants:**

Sixty-nine participants (mean age: 66.9 years; 50.7% female) were recruited from ophthalmology clinics at the University of California, San Francisco, California, United States. Eyes were grouped clinically into perimetric glaucoma (62 eyes), preperimetric glaucoma (31 eyes), and glaucoma suspect or ocular hypertension (41 eyes).

**Methods:**

Increment and decrement stimuli were tested at 12 locations per eye using the VVP system.

**Main Outcome Measures:**

Contrast sensitivities (CSs) were recorded and mean CS was computed and compared between VVP and conventional perimetry (Humphrey Visual Field [HVF]). Correlations between VVP and HVF results were computed, and statistical analyses were conducted using cluster bootstrapping to account for intereye correlations.

**Results:**

Perimetric glaucoma eyes had stronger correlation of CS between VVP and HVF tests compared to preperimetric glaucoma and glaucoma suspect or ocular hypertension eyes. Across all groups, decrement testing generally showed higher correlations than increment testing (perimetric: 0.48 [increment] vs. 0.61 [decrement]; preperimetric: 0.24 vs. 0.37; glaucoma suspect: 0.35 vs. 0.36). In perimetric glaucoma, particularly in moderate to severe cases, there was a significantly greater CS to decrement stimuli compared to increment stimuli (–1.46 decibels [dB] [95% confidence interval [CI]: –2.59, –0.30]). Preperimetric glaucoma eyes had significantly higher CS to increment stimuli than to decrement stimuli (+0.86 dB [95% CI: 0.11, 1.67]). Throughout all clinical subtypes, areas of the visual field with higher contrast thresholds (lower sensitivity) showed greater sensitivity to increment stimuli, whereas areas with lower contrast thresholds (higher sensitivity) showed greater sensitivity to decrement stimuli (perimetric: m = 0.63, R = 0.71; preperimetric: m = 0.50, R = 0.6; glaucoma suspect: m = 0.45, R = 0.51; all *P* < 0.01). Additionally, peripheral points generally exhibited lower CS than central points in both increment and decrement testing in all groups (all *P* < 0.05).

**Conclusions:**

We found significant differences in how preperimetric and perimetric glaucoma eyes respond to varying stimuli. Preperimetric eyes were better at detecting increment stimuli, while perimetric eyes were better at detecting decrement stimuli. Our results confirm earlier studies reporting greater OFF-pathway vulnerability in early glaucoma but suggest a shift to ON-pathway vulnerability in severe glaucoma.

**Financial Disclosure(s):**

Proprietary or commercial disclosure may be found in the Footnotes and Disclosures at the end of this article.

The basic concept of clinical perimetry is to measure responses to a small target against a uniform background across multiple locations in a field of view. However, many variations have been used over the years to improve perimetry as an ophthalmic diagnostic tool. One important application of perimetry is in the diagnosis and clinical evaluation of glaucoma, a complex neurodegenerative disease characterized by progressive visual field defects because of the death of the retinal ganglion cells (RGCs) that comprise the optic nerve.^1^ The American Academy of Ophthalmology's report of assessment of visual function in glaucoma^2^ describes static automated perimetry with white-on-white stimuli using the Swedish Interactive Threshold^3^^,^^4^ Algorithm (SITA) as the current benchmark for detecting visual function loss in glaucoma; however, it is known that significant damage to the optic disc can occur before visual field defects are detectable.^5^^,^^6^ Specifically, up to 40% of RGCs can be lost before these functional defects are observed,^7^^,^^8^ and glaucomatous nerve fiber layer loss can be measured by OCT up to 6 years before visual field defects become apparent,^9^, ^10^, ^11^ which underscores the need for more sensitive perimetry methods.

To address this need, various strategies have been developed to detect visual field defects earlier. Methods such as blue-on-yellow or short-wavelength automated perimetry,^12^, ^13^, ^14^, ^15^ flicker perimetry,^10^^,^^16^, ^17^, ^18^ frequency doubling perimetry,^19^^,^^20^ motion perimetry,^21^ high-pass resolution perimetry,^22^ rarebit perimetry,^20^^,^^23^ and pulsar perimetry^24^, ^25^, ^26^, ^27^ have been developed in attempts to detect glaucoma at earlier stages and with greater sensitivity and specificity. Many of these strategies are designed to probe specific RGC types: wavelength automated perimetry detects the small bistratified RGCs in the koniocellular pathway; high-pass perimetry is specific for the midget RGCs in the parvocellular pathway; and flicker perimetry, motion perimetry, and frequency doubling perimetry are designed to interrogate the parasol RGCs in the magnocellular pathway.^28^ This focus on specific RGC types in glaucoma is supported by animal models of experimental glaucoma, which suggest that the different types of RGCs may vary in their vulnerability to elevated intraocular pressure (IOP).^29^, ^30^, ^31^, ^32^, ^33^

One paradigm of RGCs with potentially differential susceptibility in glaucoma are the ON vs OFF RGCs, where ON RGCs depolarize in response to the onset of light and OFF RGCs hyperpolarize to onset of light but depolarize to the offset of light (or onset of dark).^34^ Although the biological basis of differential RGC susceptibility in glaucoma is still unknown, noninvasive assays of ON vs OFF pathway function may prove to be a meaningful path forward toward earlier glaucoma detection and diagnosis. Animal studies have generally found that the ON RGCs are more resilient than OFF RGCs in glaucoma models,^35^, ^36^, ^37^, ^38^, ^39^, ^40^ although there have also been studies that suggest that differential susceptibility does not follow a dichotomous ON vs OFF RGC paradigm.^41^, ^42^, ^43^, ^44^ In human glaucoma, studies using pattern electroretinogram (ERG),^45^ photopic negative response full-field ERG,^46^^,^^47^ flicker visual evoked potential,^48^^,^^49^ sinusoidal flicker ERG,^50^^,^^51^ steady-state visual evoked potentials to increment and decrement sawtooth stimuli,^52^ computerized threshold testing with light offsets on cathode ray tubes,^53^ and computer assisted moving eye campimeter^54^ have demonstrated increased susceptibility of the OFF pathway. However, there have also been studies in human glaucoma that show no difference in foveal contrast processing of increment or decrement targets,^55^ and that dark targets are detected faster and more accurately than light targets.^56^ One limitation of previous studies is that the vast majority of interrogations of ON-/OFF-pathway sensitivity have been conducted in response to full-field stimuli, and the one perimetric study was limited to perimetric glaucoma eyes with a mean deviation of –5.2 decibels [dB].^54^ To date, there have been no studies investigating increment and decrement stimuli across different stages of glaucoma using virtual reality (VR)-based perimetry.

Virtual reality technology provides a promising alternative approach to conventional perimetry methods, such as the Humphrey Visual Field (HVF) Analyzer and the Goldmann Perimeter, which require expensive devices, have high patient fatigue, and a physically rigid testing environment.^57^, ^58^, ^59^ Distinct advantages of using a VR testing format include the enclosed nature of the headset, which can minimize distractions and external light interference when assessing the ON and OFF visual pathways; the requirement for the patient to suppress their foveation reflex (specific to Vivid Vision Perimetry [VVP])^60^; the lack of a need for a trained technician to monitor compliance; and flexibility in the testing environment, including at-home testing.^61^ Our current study utilizes a previously validated VR-based perimetric system VVP^61^^,^^62^ for the following aims: interrogate both ON and OFF retinal pathways using increment and decrement stimuli in participants with perimetric glaucoma, preperimetric glaucoma, and glaucoma suspect or ocular hypertension; and quantify agreement between increment and decrement stimuli testing by VVP with conventional static automated perimetry.

## Methods

Participants with perimetric glaucoma, preperimetric glaucoma, and glaucoma suspect or ocular hypertension diagnoses were enrolled from ophthalmology clinics at the University of California, San Francisco. Informed consent was obtained from all participants in the clinic. All methods were approved by the University of California, San Francisco, Institutional Review Board (IRB approval no: 16-20210), and all research adhered to the tenets of the Declaration of Helsinki and the Health Insurance Portability and Accountability Act.

### Participant Characteristics

Participants aged 18 to 85 years with a diagnosis of glaucoma or glaucoma suspect or ocular hypertension were included in the study. Participants with perimetric glaucoma were identified through optic nerve damage indicated by structural abnormalities in either the optic disc or retinal nerve fiber layer (RNFL) and visual field abnormalities consistent with RNFL damage on HVF testing. Abnormal disc appearance was defined by neuroretinal rim thinning, localized or diffuse RNFL defects, disc hemorrhages, or progressive narrowing of the neuroretinal rim with increased cupping, detected through slit lamp biomicroscopy with a handheld lens or spectral-domain OCT imaging. Visual field defects were defined as the repeatable and reproducible presence of glaucomatous visual field loss (eg, nasal step, arcuate scotoma, paracentral defect), and visual field defects corresponded to structural defects measured by spectral-domain-OCT as determined by a glaucoma specialist [Y.O.]. Participants with preperimetric glaucoma were identified through the same parameters as for perimetric glaucoma, with the added criteria that they did not have persistent visual field defects on HVF and the Glaucoma Hemifield Test was “within normal limits.” Ocular hypertension was diagnosed by the presence of consistently elevated IOP (above 21 mmHg) without evidence of optic nerve cupping or RNFL thinning, and glaucoma suspect eyes were defined as suspicious optic nerve or RNFL without visual field defects and with no treatment history for ocular hypertension. None of the participants had undergone prior testing with the increment or decrement VVP test. All participants were also tested with the HVF (24-2 SITA Standard). Participants with refractive errors were instructed to wear their distance-corrected glasses when taking the test.

The exclusion criteria included participants who had a best-corrected visual acuity worse than 20/80, refractive errors greater than ±6.0 D, significant cataracts, or concurrent retinal diseases such as retinal vein occlusion, wet age-related macular degeneration, or proliferative diabetic retinopathy. Additionally, individuals with a history of epilepsy, active facial infections or acne or rosacea, active cold or cough, and issues with neck strain or head movements were also excluded from the study.

### VVP Test Mechanics

**Figure 1:**
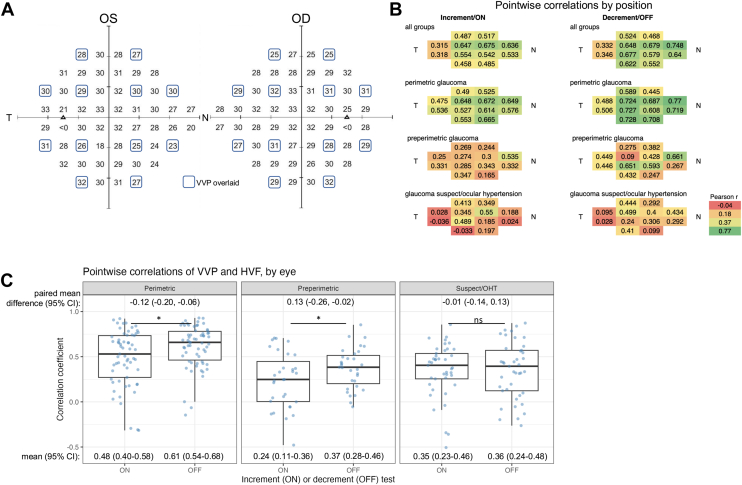
Comparison of VVP and HVF sensitivities by testing position and eye. **A,** Overview of where VVP test locations are shared with a 24-2 HVF (placeholder values are also shown). **B**, Pearson correlation coefficients between the VVP contrast sensitivity and HFA retinal sensitivity value were calculated for each test position across all eyes. Both left and right eyes were collapsed into a single analysis. N and T represent the Nasal and Temporal edges of the visual field. Correlations were calculated separately for increment and decrement testing and for both all participant groups and within each clinical subtype. **C,** Pearson correlation coefficients between VVP contrast sensitivity and HVF retinal sensitivity values were calculated for each eye, and the distribution of coefficients is depicted graphically across clinical subtypes (perimetric glaucoma, preperimetric glaucoma, and GS or OHT). Bootstrapped estimates of the means and paired mean differences are labeled; 95% CIs derived from the bootstrapped estimates were used to determine significance. ∗*P* < 0.05. CI = confidence interval; GS = glaucoma suspect; HVF = Humphrey visual field; ns = not significant; OD = right eye; OHT = ocular hypertension; OS = left eye; VVP = Virtual Vision Perimetry.

Vivid Vision Perimetry was used to administer separate increment and decrement tests using a VR headset. Each test included stimuli at 12 locations per eye (Fig 1A). The 12 locations were chosen to balance selected points across the same 52 test locations that HVF tests while balancing test duration. Participants took the increment and decrement tests sequentially, with the starting condition counterbalanced across participants, alternating by enrollment order. Within a test, stimuli alternated randomly between the 2 eyes. Contrast at the 12 test locations was controlled by a 1-up, 1-down staircase, changing by a factor of 2 or 4 between steps. All stimuli were shown on a white background (26 cd/m^2^) and were discs with a square wave spatial profile to which a raised cosine blur skirt was applied. Stimuli were 1.72° in diameter at half-height (Goldmann Size V). A stimulus of Goldmann size V was selected to accommodate the limited dynamic range of the VR headset (see Appendix A, available at www.ophthalmologyscience.org) and to reduce test-retest variability in areas of glaucomatous visual field loss.^63^^,^^64^ Stimulus duration was 0.30 seconds (square wave pulse) and was chosen to increase stimulus energy and dynamic range while remaining too brief to permit visual search for low-contrast stimuli. To assure correct stimulus contrast, headset luminance was calibrated using an ILT2400/SED033-Y4 spot photometer (International Light Technologies Inc). Stimulus contrast was transformed to match HVF sensitivity (Appendix A). Test stimuli were presented 4 times per location per test (similar to algorithms such as SITA^65^), with location chosen at random on each trial. Thus, the total number of stimuli per test was 2 eyes × 12 locations per eye × 4 stimuli per location = 96 stimuli per test.

At the end of the test, threshold was computed at each tested location by fitting a psychometric function (cumulative normal) to the data. Because all contrast staircases started at the same contrast and there were only 4 stimuli per location, threshold estimates for a given type of test (increment or decrement) took on 1 of 11 discrete values.

Contrast was Weber contrast (increment or decrement in luminance, divided by the background luminance) and contrast sensitivity (CS) was the reciprocal of the contrast threshold (ie, the additive inverse of contrast threshold when expressed in dB). Contrast sensitivity was computed for increment and decrement stimuli tests in all test locations per eye. The mean CS (MCS) was the mean sensitivity across the 12 test locations. Summary statistics describing reliability criteria (median response time, median fixation time, and guess rate) are included in Table S1 (available at www.ophthalmologyscience.org).

### Statistical Analysis

The statistical tests used to compare demographic and ocular characteristics between different patient groups were as follows: chi-square test of independence (gender), Fisher exact test (race and ethnicity), and 1-way analysis of variance (all other variables). Cluster bootstrapping for statistical analysis was conducted to account for intereye correlations.^66^ Briefly, each bootstrapped sample contained a random sample with replacement of the total number of participants in each participant group; if there were 2 eyes for a given participant with the ocular disease of interest, a random eye was selected. The desired statistic was computed using this bootstrapped sample and repeated 1000 times. The median value of this distribution serves as the estimate of the desired statistic and a 95% confidence interval (CI) was determined through the 2.5th and 97.5th percentiles of the ordered distribution. Pearson correlations were calculated for individual CS values from VVP and the retinal sensitivity values from HVF. Welch t-test was used to compare central and peripheral CS values.

## Results

### Study Population Characteristics

Sixty-nine of the 87 participants completed testing (mean [standard deviation] age was 66.9 [10.4] years; 50.7% female). Participants performed 1 increment test (mean [standard deviation]: 335.7 s [64.1 s]) and 1 decrement test (352.9 s [85.6 s]), lasting a total of ∼12 minutes. Reasons for incompletion included failure to complete the VVP tutorial because of inability to complete fixation task (n = 13), fatigue or heavy headset (n = 3), double vision (n = 1), and blue or green color deficiency (n = 1). One enrolled participant was clinically excluded because of central visual field defects from coexisting age-related macular degeneration. The remaining eyes (68 right eyes [ODs], 66 left eyes [OSs]) were grouped clinically into: perimetric glaucoma (62 eyes), preperimetric glaucoma (31 eyes), and glaucoma suspect or ocular hypertension (41 eyes). Demographic and clinical characteristics of study participants are summarized in Table 1. No significant differences were found between groups except for refractive error, HVF mean deviation, HVF pattern standard deviation, mean RNFL thickness, and mean ganglion cell complex thickness.

**Table 1:** Patient Demographics and Ocular Characteristics

Demographics and Ocular Characteristics	Perimetric Glaucoma (n = 39; 62 Eyes)	Preperimetric Glaucoma (n = 23; 31 Eyes)	Glaucoma Suspect or Ocular Hypertension (n = 22; 41 Eyes)	*P* Value
Age (yrs; mean ± SD)	67.3 ± 10.4	65.6 ± 13.5	67.2 ± 6.9	0.72
Gender (% female)	38.7	74.2	48.8	0.005
Race (%)				0.30
White	29 (46.8%)	13 (41.9%)	18 (43.9%)	
Asian	22 (35.5%)	12 (38.7%)	8 (19.5%)	
Black	5 (8.1%)	3 (9.7%)	6 (14.6%)	
Other	5 (8.1%)	2 (6.5%)	7 (17.1%)	
Unknown	1 (1.6%)	1 (3.2%)	0 (0%)	
Pacific Islander	0 (0%)	0 (0%)	2 (4.9%)	
Ethnicity (%)				0.10
Not Hispanic or Latino	62 (100%)	29 (93.5%)	39 (95.1%)	
Hispanic or Latino	0 (0%)	2 (6.5%)	2 (4.9%)	
Best-corrected visual acuity (LogMAR; mean ± SD)	0.16 ± 0.19	0.12 ± 0.15	0.16 ± 0.19	0.55
Refractive error in spherical equivalents (D; mean ± SD)	–2.25 ± 3.04	–2.02 ± 2.6	–0.2 ± 3.58	0.004
Intraocular pressure (mmHg; mean ± SD)	15.4 ± 3.3	17.2 ± 3.6	17 ± 4.9	0.05
HVF mean deviation (dB; mean ± SD)	–5.0 ± 4.5	–0.3 ± 2.5	–0.2 ± 2.2	<0.001
HVF pattern standard deviation (dB; mean ± SD)	6.7 ± 3.9	1.8 ± 0.5	2 ± 1.1	<0.001
SD-OCT mean RNFL (μm; mean ± SD)	71.5 ± 9.8	86.8 ± 12.1	89.1 ± 10.9	<0.001
SD-OCT mean GCC (μm; mean ± SD)	76.2 ± 9.4	87 ± 9	89 ± 9.5	<0.001

dB = decibel; GCC = ganglion cell complex; HVF = Humphrey visual field; LogMAR = logarithm of the minimum angle of resolution; RNFL = retinal nerve fiber layer; SD = standard deviation; SD-OCT = spectral-domain OCT.

The mean ± standard deviation and number of observations (proportion) were reported.

Statistical tests: for gender, chi-square test of independence was used. For race and ethnicity, Fisher exact test was used. For all other variables, 1-way analysis of variance was used.

### Comparison of VVP to HVF Testing

We compared the results from VVP increment and decrement testing with conventional HVF white-on-white testing. The 12 points tested by VVP coincide with 12 of the points tested by HVF (Fig 1A). We calculated the correlation for each individual VVP test position across all eyes between the VVP CS and the HVF retinal sensitivity values at these specific test locations (Fig 1B). Overall, perimetric glaucoma in both increment and decrement testing provided stronger correlations than preperimetric glaucoma, which were stronger than for glaucoma suspect/ocular hypertension. Decrement testing had higher correlations in general than increment testing between groups.

We then calculated correlation coefficients at a per-eye level with the 12 tested points in VVP and the corresponding HVF points (Fig 1C) and then used a bootstrapped estimate of the mean correlation coefficient that accounted for intereye correlation to compare between groups. In both increment and decrement testing, perimetric glaucoma eyes (increment: 0.48, 95% CI: 0.40–0.58; decrement: 0.61, 95% CI: 0.54–0.68) had a higher mean correlation coefficient (R) than preperimetric glaucoma (increment: 0.24, 95% CI 0.11–0.36; decrement: 0.37, 95% CI: 0.28–0.46) and glaucoma suspect or ocular hypertension eyes (increment: 0.35, 95% CI: 0.23–0.46; decrement: 0.36, 95% CI: 0.24–0.48). Increment testing had a lower mean correlation than in decrement testing in the glaucoma or ocular hypertension eyes (paired mean difference in correlation coefficient: –0.12; 95% CI: –0.20 to –0.06) and preperimetric glaucoma (–0.13; 95% CI: –0.26 to –0.02) but not in glaucoma suspect/ocular hypertension (–0.01; 95% CI: –0.14 to 0.13).

In summary, perimetric glaucoma eyes had a higher correlation of CS between VVP and HVF testing compared to preperimetric glaucoma and glaucoma suspect/ocular hypertension eyes, with decrement testing showing generally higher correlations than increment testing across groups.

### Comparison of the Mean CS between Increment and Decrement Stimuli

**Figure 2:**
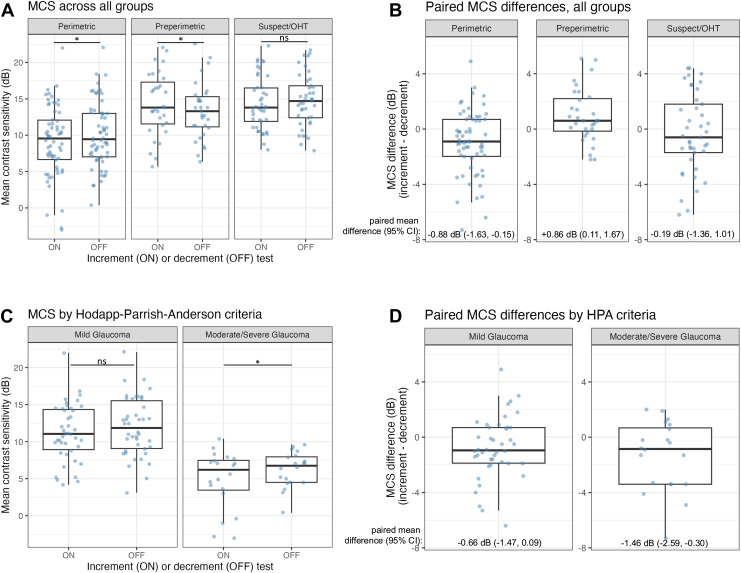
Distribution and paired differences of mean contrast sensitivity across clinical subtypes and glaucoma severity levels. **A,** Distribution of MCS (dB) in both increment (ON) and decrement (OFF) stimuli testing across clinical subtypes (perimetric glaucoma, preperimetric glaucoma, and GS or OHT). Significance was determined using the bootstrapped estimate of the 95% CIs of the paired mean differences. **B,** Distribution of paired differences in mean contrast sensitivity from **A** (dB; increment MCS minus decrement MCS) across clinical subtypes, with bootstrapped estimates (accounting for intereye correlation) of paired mean difference and 95% CI labeled. **C,** Glaucoma eyes were divided by Hodapp–Parrish–Anderson scoring from HFA testing into mild and moderate or severe. Significance was determined as described for **A**. **D,** Distribution of paired differences in mean contrast sensitivity (dB) from C, calculated as previously described in B. CI = confidence interval; dB = decibel; GS = glaucoma suspect; HPA = Hodapp–Parrish–Anderson; MCS = mean contrast sensitivity; ns = not significant; OHT = ocular hypertension. ∗ statistically significant, bootstrapped estimate of 95% CI are not overlapping.

The MCS of all 12 points tested for each eye was calculated and compared between increment and decrement stimuli and across groups. The estimated paired mean differences by cluster bootstrap between increment and decrement (increment minus decrement) testing were calculated. A positive mean difference indicates that the eye has a higher CS in response to increment stimuli versus decrement stimuli, and a negative mean difference indicates that the eye has a higher CS in response to decrement stimuli versus increment stimuli. There is a statistically significant negative difference in perimetric glaucoma (–0.88 dB; 95% CI: –1.63 dB to –0.15 dB) and a positive difference in preperimetric glaucoma (difference of +0.86 dB; 95% CI: 0.11 dB to 1.67 dB) but not in glaucoma suspect or ocular hypertension (–0.19 dB; 95% CI: –1.36 dB to 1.01 dB) (Fig 2A, B). After classifying perimetric glaucoma participants by Hodapp–Parrish–Anderson criteria,^67^ there is a statistically significant negative paired mean difference between increment and decrement stimuli in the moderate-severe eyes (–1.46 dB, 95% CI: –2.59 to –0.30) but not in the mild eyes (–0.66 dB, 95% CI: –1.47 dB to 0.09 dB) (Fig 2C, D). In summary, there was a significant higher CS to decrement (vs. increment) stimuli in perimetric glaucoma, especially in moderate-severe glaucoma and a significantly higher CS to increment (vs. decrement) stimuli in preperimetric glaucoma.

### Pointwise Responses to Increment and Decrement Stimuli

**Figure 3:**
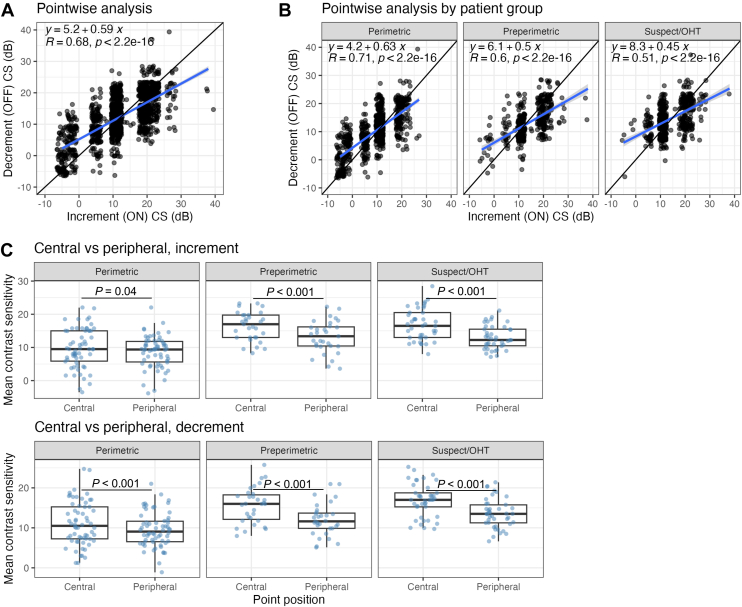
Pointwise analysis and distribution of contrast sensitivity across clinical subtypes and test locations. **A,** Pointwise analysis across all points. All VVP test locations decrement CS and increment CS are plotted. Linear regression equations and Pearson correlation R and *P* values are displayed in inset. Because of the discrete distribution of contrast sensitivity values, a jitter was added in both x and y dimensions to better visualize point density. **B,** Same as in A, after separating into clinical subtypes (perimetric glaucoma, preperimetric glaucoma, and GS or OHT). **C,** Distribution of the mean CS of the central 4 and mean CS of the peripheral 8 points for each eye, across clinical subtypes and in both increment and decrement testing. Welch paired t-test was used to compare between central and peripheral points. CS = contrast sensitivity; GS = glaucoma suspect; OHT = ocular hypertension.

A pointwise analysis was conducted where all 12 points tested for each eye were pooled and increment and decrement CS values were plotted against each other. All linear regression slopes were <1, and all correlation values were significant in the entire study population (m = 0.59, R = 0.68, *P* < 0.01) and in perimetric glaucoma (m = 0.63, R = 0.71, *P* < 0.01), preperimetric glaucoma (m = 0.50, R = 0.6, *P* < 0.01), and glaucoma suspect or ocular hypertension (m = 0.45, R = 0.51, *P* < 0.01) (Fig 3A, B), which suggests that areas with higher contrast thresholds have higher increment CSs, whereas areas with lower contrast thresholds have higher decrement CSs. The MCS of the 8 peripherally tested points is significantly lower than the MCS of the 4 central points in all 3 participant groups and in both increment and decrement testing (all *P* < 0.001), with the exception of increment testing in perimetric glaucoma (*P* = 0.04), the (Fig 3C). In summary, areas of the visual field with higher contrast thresholds have higher increment (vs. decrement) sensitivities and areas with lower contrast thresholds have higher decrement (vs. increment) sensitivities across all clinical subtypes, and, in general, peripheral points have lower CS than central points in both increment and decrement testing.

## Discussion

In this study, we interrogated ON and OFF pathways in glaucoma through a VR-based perimetric approach where increment and decrement testing was performed across multiple points in the visual field. We assayed 12 unique points across the visual field and use individual CS as an output. Directly comparing increment and decrement testing by VVP to conventional HVF revealed that decrement testing by VVP generally has a higher pointwise correlation to HVF as compared to increment testing, particularly in perimetric glaucoma participants. However, there were no differences between increment and decrement testing in the correlation between the derived aggregate values of MCS and HVF mean deviation. We did see an expected higher correlation in perimetric glaucoma as compared to preperimetric glaucoma and glaucoma suspect or ocular hypertension, possibly because of having a larger dynamic range as opposed to clustering around high MCS and MD values in preperimetric and glaucoma suspect or ocular hypertension groups. However, differences in the stimuli presentation and characteristics make it difficult to directly compare VVP CSs with HVF sensitivity values in decibels.

We also identified differences in response to increment and decrement stimuli in preperimetric and perimetric glaucoma but not glaucoma suspect or ocular hypertension participants. There was a statistically significant negative mean difference in MCS between increment and decrement testing in perimetric glaucoma participants, which suggests that perimetric glaucoma eyes had higher CSs in response to decrement stimuli than increment stimuli. Conversely in preperimetric glaucoma, there was a statistically significant positive mean difference in MCS between increment and decrement testing, which suggests that preperimetric eyes had higher CSs in response to increment stimuli than decrement stimuli. When the perimetric glaucoma eyes were reclassified by Hodapp–Parrish–Anderson criteria, moderate-severe eyes but not mild eyes had statistically significant negative MCS difference. This suggests that within perimetric glaucoma, the eyes which have the worse mean deviation are the ones which have higher CSs in decrement than increment stimuli. Importantly, the raw MCS values in both increment and decrement testing were decreased in the perimetric glaucoma group compared to the preperimetric glaucoma group, which is consistent with the expectation that regardless of stimuli type, perimetric glaucoma eyes have worse vision than preperimetric glaucoma eyes. These findings may be explained by known retinal physiology: ON and OFF visual pathways, which respond preferentially to increments and decrements in light, respectively, may be differentially affected at various stages of glaucoma. It is known that there is greater OFF ganglion cell vulnerability early in the disease process seen in experimental models of glaucoma as well as clinical glaucoma.^35^, ^36^, ^37^, ^38^, ^39^, ^40^^,^^45^, ^46^, ^47^, ^48^, ^49^, ^50^^,^^52^ In our study, preperimetric glaucoma eyes showed higher sensitivity to increment stimuli, which may reflect early OFF-pathway dysfunction. Conversely, in moderate-to-severe perimetric glaucoma, we observed higher sensitivity to decrement sensitivity (relative preservation of decrement sensitivity), possibly because of subsequent ON-pathway involvement as the disease progresses. Future experimental studies, perhaps in experimental or animal models of glaucoma, is necessary to test this hypothesis directly.

The pointwise analysis of individual CS values highlighted differences seen in response to increment vs. decrement stimuli. At points with higher contrast thresholds, defined by higher values of both increment and decrement CS, we found higher increment CS values as compared to decrement CS values. In contrast, points with lower contrast thresholds, defined by areas with both lower increment and decrement CS, had higher decrement CS values as compared to increment CS values. These results were consistent across all 3 groups. One model to explain the results of increased OFF-pathway vulnerability in preperimetric glaucoma vs. increased ON-pathway vulnerability in perimetric glaucoma (Fig 2) is that most of the tested points fall in high vision areas in preperimetric glaucoma eyes, which have higher increment CS values compared to decrement CS values and therefore increased OFF-pathway vulnerability. In contrast, in perimetric glaucoma eyes most tested points fell in low vision areas, which had higher decrement CS values compared to increment CS values and therefore increased ON-pathway vulnerability. Also of note is that, in glaucoma suspect or ocular hypertension participants, the trend of higher CS values having higher increment CS as compared to decrement CS persisted, although perhaps these tested points were too sparse or there is high participant-to-participant variability, which led to no significant difference in the MCS between increment and decrement tests in this group. We also observed significantly decreased CS values in peripheral VVP tested points compared to central tested points in all groups and in both increment and decrement testing, which is consistent with peripheral vision loss preceding central vision loss in glaucoma.^68^ In addition, in our study the 2 temporal points tested generally had lower pointwise correlations than the 2 nasal points, which is consistent with prior studies on different portable perimeters also demonstrating the same trend of regional discrepancies, with the temporal fields having the lowest correlation with HVF testing.^69^^,^^70^ We also note that, although our VVP system reveals differences in CS, no differences were seen in response time to increment and decrement stimuli in perimetric and preperimetric glaucoma (data not shown), which is consistent with previously presented findings.^71^

This study had several important limitations. We enrolled participants that were clinically defined as glaucoma suspect or ocular hypertension, preperimetric glaucoma, and perimetric glaucoma but there was substantial variation and overlap among groups in terms of CS values, MCS values, and HVF mean deviations. There were a substantial number of tested points with CS values <15 dB in the glaucoma suspect/ocular hypertension group. Although we have included the spectrum of glaucoma progression from suspect or ocular hypertension to preperimetric to perimetric glaucoma, future studies should include a true normal vision control group to better define normal vision responses to these increment and decrement stimuli in the context of our test. At the moment, it is not clear whether the preperimetric and glaucoma suspect groups have normal sensitivity to responses or also have deficits. However, prior studies in participants with normal vision using full-field sawtooth increment and decrement stimuli, have described increased steady-state visually evoked potential amplitudes in response to contrast decrements when compared to contrast increments,^72^ as well as improved decrement CS compared to increment CS,^73^ and we would expect to find similar results if we applied our assay to normal vision participants. Another limitation is that we use square wave stimuli, as opposed to sawtooth stimuli. Although they provide qualitatively similar results, sawtooth stimuli isolate ON and OFF responses more distinctly while minimizing adaptation effects when compared to square wave stimuli.^74^ Test-retest variability could not be directly measured in our study as the participants only took each test once but was estimated to be similar to HVF (SITA Fast) because we designed our test to present a similar number of stimuli at each location; this unquantified variability likely contributed to overall variance and may have widened CIs and reduced statistical power. In addition, we were also limited from a technical standpoint of reaching a “floor” of measurable CS, in that we cannot physically display a darker or brighter stimulus on the headset. We also only test 12 locations of the visual field, in contrast to the 54 points tested by the standard HVF 24-2, which may not reflect an even distribution of low vision and high vision locations across participants. The 12 locations were selected to provide broad coverage of the tested visual field, balancing spatial sampling with test duration constraints. Although peripheral points typically exhibit greater variability, their inclusion enabled exploration of CS across a range of eccentricities. Future studies may consider alternative point selections (eg, more nasal points) to increase sensitivity to early glaucomatous changes. Given the known effects of axial myopia on RNFL thickness, it is possible that some individuals classified as preperimetric or perimetric glaucoma may have had myopic RNFL thinning rather than true glaucomatous damage. To address this, we excluded high myopes from this study (>6.0 D), but the perimetric and preperimetric group mean refractive errors were still in the low myopia range (–2.25 D and –2.02 D, respectively), whereas the mean glaucoma suspect group's error was close to plano (–0.2 D). Future studies should incorporate axial length or refractive error matching to further reduce the risk of misclassification. Although we enrolled participants across the spectrum of glaucoma stages, we did not power the study to examine differences among these groups.

In this study, we evaluated responses to increment and decrement stimuli in individuals with varying stages of glaucoma using a VR-based perimetric approach. We noted significant differences in how preperimetric and perimetric glaucoma eyes responded to these stimuli, with preperimetric glaucoma eyes being better at detecting increment than decrement stimuli, and perimetric glaucoma eyes being better at detecting decrement than increment stimuli. This suggests a potential shift in pathway vulnerability from OFF to ON channels as glaucoma progresses. Our results confirm previous studies showing greater OFF-pathway vulnerability in early glaucoma stages^45^, ^46^, ^47^, ^48^, ^49^, ^50^^,^^52^, ^53^, ^54^^,^^75^ but also suggest that, in severe glaucoma, there is a shift to where the ON pathway becomes relatively more vulnerable.

Additionally, in our study, we show that in all stages of glaucoma, low vision areas generally exhibit relatively increased ON-pathway vulnerability, whereas high vision areas exhibit relatively increased OFF-pathway vulnerability. These observations could be consistent with prior studies of the ON and OFF pathway^45^, ^46^, ^47^, ^48^, ^49^, ^50^^,^^52^ that generally show overall increased OFF-pathway vulnerability because they have all used full-field stimuli and were therefore likely biased toward areas of high vision. However, our use of a VR-based perimetric approach provides a more localized analysis of ON and OFF pathways across multiple visual field points, revealing nuanced differences in pathway vulnerabilities that may not be apparent with full-field stimuli.

These findings motivate future electrophysiologic studies such as multifocal ERG or visual evoked potential to further investigate these selective vulnerabilities of ON and OFF pathways in high and low vision areas. Our study both corroborates existing research on OFF-pathway vulnerability but also introduces a new perspective on ON-pathway involvement in advanced glaucoma, thus motivating further research into targeted electrophysiologic assessments in glaucoma patients and design of stimulus strategies to monitor glaucoma progression.
