# Supplementary material for: Evaluating Increment and Decrement Stimuli Responses in Patients with Glaucoma Using Virtual Reality–Based Perimetry
Source: Ophthalmol Sci. 2025 Aug 28;6(1):100929. doi: 10.1016/j.xops.2025.100929 (PMC12548083; doi:10.1016/j.xops.2025.100929)
Supplement: Appendix A [file mmc1.docx]

**Appendix A:** Linear transformation of Vivid Vision Perimetry (VVP) physical contrast to familiar dB for Goldmann Size III stimuli

VR headsets have lower luminance than conventional perimeters, so display parameters must be chosen carefully in order for VR sensitivity measurements to be comparable with conventional measurements. In this study, headsets had a maximum luminance of 95 cd/m^2^ and the background luminance was 26 cd/m^2^, so the maximum physical contrasts for increments and decrements were severely constrained. Nevertheless, we found that by using Size V stimuli instead of Size III, and a slightly longer display duration of 300 msec instead of 200 msec, neither of our two physical contrast thresholds were truncated when compared with the HFA sensitivity measurements at corresponding retinal locations. For convenience, we have reported sensitivity measurements for VVP in dB units that were scaled to match the HFA measurements. The actual linear transformation used was dB_VVP_ = -1.60 + (2.26)(10×log_10_(C_thresh_)) where C_thresh_ is the contrast threshold of the stimulus. A linear transformation does not affect the analyses in this paper, because effects and measurement noises scale equally, and none of the analyses required the combination of measurements from the two devices into a single statistic.
