# Supplementary material for: Evaluating Increment and Decrement Stimuli Responses in Patients with Glaucoma Using Virtual Reality–Based Perimetry
Source: Ophthalmol Sci. 2025 Aug 28;6(1):100929. doi: 10.1016/j.xops.2025.100929 (PMC12548083; doi:10.1016/j.xops.2025.100929)
Supplement: Table S1 [file mmc2.docx]

**Supplemental Table 1: Reliability criteria summary statistics**

| **Test** | **Patient Group** | **Response time (sec)** | **Fixation Time (sec)** | **Guess Rate** |
| --- | --- | --- | --- | --- |
| Decrement | Perimetric glaucoma | 723.2 ± 113.7 | 1230.4 ± 541.4 | 0.14 ± 0.23 |
| Decrement | Preperimetric glaucoma | 716.9 ± 91.9 | 1045.3 ± 146.7 | 0.05 ± 0.06 |
| Decrement | Glaucoma suspect | 706.6 ± 93.0 | 1144.9 ± 195.3 | 0.06 ± 0.06 |
| Increment | Perimetric glaucoma | 705.7 ± 120.5 | 1215.2 ± 425.4 | 0.16 ± 0.24 |
| Increment | Preperimetric glaucoma | 700.4 ± 124.2 | 1125.5 ± 400.9 | 0.12 ± 0.17 |
| Increment | Glaucoma suspect | 683.5 ± 91.0 | 1115.9 ± 156.4 | 0.06 ± 0.08 |

Mean±SD is reported. Two-way ANOVA was performed on *test*, *patient group*, and the interaction *test:patient group* for each reliability criteria variable. The p-value for *patient group* on *fixation time* was *p*=0.042. The p-value for *patient group* on variable *guess rate* was *p*<0.001. All other p-values were *p*>0.05.
